# The impact of perinatal inflammation on the electroencephalogram in preterm infants: a systematic review

**DOI:** 10.1038/s41390-022-02038-3

**Published:** 2022-04-01

**Authors:** Antoine Giraud, Carol M. Stephens, Geraldine B. Boylan, Brian H. Walsh

**Affiliations:** 1 INFANT Research Centre, University College Cork, Cork, Ireland; 2 INSERM, U1059 SAINBIOSE, Université Jean Monnet, Saint-Étienne, France; 3 Department of Paediatrics and Child Health, University College Cork, Cork, Ireland; 4 Department of Neonatology, Cork University Maternity Hospital, Cork, Ireland

## Abstract

**Background:**

To summarise the association between perinatal inflammation (PI) exposure and electroencephalography (EEG) features in preterm infants.

**Methods:**

This systematic review included clinical studies of preterm infants born <37 weeks of gestational age (GA), who had both a PI exposure and an EEG assessment performed during the neonatal period. Studies were identified from Medline and Embase databases on the 15th of September 2021. PI was defined by histological chorioamnionitis, clinical chorioamnionitis, or early-onset neonatal infection (EONI). The risk of bias in included studies was assessed using the Joanna Briggs Institute (JBI) appraisal tool. A narrative approach was used to synthesise results. This review followed the Preferred Reporting Items for Systematic reviews and Meta-Analyses (PRISMA) 2020 statement.

**Results:**

Two cross-sectional studies enrolling 130 preterm children born <32 weeks of GA assessed with one-channel amplitude-integrated EEG (aEEG) during the first four days of life were included. A PI exposure was described in 39 (30%) infants and was associated with a decrease in amplitude and a reduced incidence of sleep-wake cycling patterns.

**Conclusion:**

These results should be interpreted with caution because of the small number of included studies and their heterogeneity. Further clinical studies evaluating the association of PI with EEG findings are needed.

**Impact:**

- A method to assess developmental trajectories following perinatal inflammation is required.
- Insufficient data exist to determine EEG features associated with perinatal inflammation.
- Further clinical studies evaluating this association are needed.

## Introduction

Preterm infants are at high risk of neurodevelopmental disabilities^1^. Early identification of developmental trajectories in preterm infants is crucial to initiate appropriate early intervention, thereby optimising future outcome^2^. Neonatal electroencephalography (EEG) provides a reliable assessment of brain activity and maturation^3,4^, which are associated with neurodevelopmental outcomes^5,6^.

Perinatal inflammation exposure in preterm infants is associated with severe neonatal brain injuries, such as grade 3 and 4 cerebral haemorrhage and cystic periventricular leukomalacia^7,8^. Perinatal inflammation is also independently associated with cerebral palsy and other neurodevelopmental impairments^9–11^.

Current markers for perinatal inflammation are unable to provide additional risk stratification for developmental outcome among these infants. This is most relevant for those without identified neonatal brain injuries, who remain at significant risk of neurodevelopmental impairments due to the perinatal inflammation exposure^11^. Additional methods of assessing the impact of perinatal inflammation on the developing brain would be of tremendous benefit. This would assist in both counselling families and ensuring that appropriate targeted services are available to maximise the infant’s developmental potential.

Preterm perinatal inflammation exposure has been shown to induce an alteration of EEG maturation in preclinical sheep models^12–18^. Identifying EEG features associated with perinatal inflammation in preterm infants will provide critical information on early brain activity, and may assist in identifying which infants exposed to perinatal inflammation are at greatest risk for altered brain growth and poor developmental trajectories^9–11^.

The aim of this systematic review is to summarise the available research on the association between perinatal inflammation exposure and EEG features in preterm infants.

## Methods

### Registration and protocol

This study was registered on the International Prospective Register of Systematic Reviews (PROSPERO) with the name “The impact of perinatal inflammation on the electroencephalogram in preterm infants: A systematic review” and registration number CRD42021284158. The review protocol can be accessed on the PROSPERO website (https://www.crd.york.ac.uk/prospero/). The protocol was conducted according to the Institute of Medicine of the National Academies Standards for Systematic Reviews^19^. The study was reported according to the Preferred Reporting Items for Systematic reviews and Meta-Analyses (PRISMA) 2020 statement^20^. Ethical approval was not required for this work as it was based on previously published data.

### Eligibility criteria

Clinical studies published in English in peer-reviewed journals reporting a perinatal inflammation exposure and a neonatal EEG assessment in children born <37 weeks of gestational age (GA) were included. Perinatal inflammation exposure was defined by histological chorioamnionitis, clinical chorioamnionitis, or early-onset neonatal infection (EONI). The definitions used for clinical chorioamnionitis and EONI are variable within the published literature, and indeed are not always defined within individual manuscripts. Therefore, all manuscripts that self-reported either clinical chorioamnionitis or EONI were considered for inclusion. Book chapters, conference papers, case reports, and review articles were excluded. Animal studies were not considered for inclusion.

### Information sources and search strategy

On the 15^th^ of September 2021, AG searched Medline (1946 – present) and Embase (1947 – present) databases, using the following search strategy: (“perinatal inflammation” OR “chorioamnionitis” OR “infection” OR “sepsis” OR “inflammation” OR “placenta”) AND (“EEG” OR “aEEG” OR “CFM” OR “electroencephalogram” OR “electroencephalography”) AND (“neonate” OR “neonatal” OR “infant” OR “newborn” OR “baby” OR “babies” OR “preterm” OR “premature” OR “prematurity”). No filters or limits were applied. A new search using the same strategy was performed on the 31^st^ of January 2022 and did not identify additional relevant records.

### Selection process

The identified records were first imported to EndNote software v20.2 (Clarivate, PA). Duplicate records, non-English languages records, conference papers records, case reports records, and review articles records were identified, manually reviewed, and then removed. Two reviewers (AG and CMS) independently screened titles and abstracts of all identified records for screening. Then, AG and CMS independently screened retrieved full-text articles for inclusion. Any disagreements during screening or inclusion stages were discussed with a third reviewer (BHW) to make the final decision.

### Data collection process

A standardised data extraction form was designed to extract study characteristics using Excel software v16.54 (Microsoft, WA). Two reviewers (AG and CMS) worked independently to extract study data. A third reviewer (BHW) reviewed data extraction and resolved any disagreements.

### Data items

The main outcome data recorded were any EEG features displayed by amplitude-integrated EEG (aEEG) or conventional EEG recording during the neonatal period. The other data included were: first author, year of publication, journal, title, type of study, study site, study period, population size, perinatal inflammation features, number of infants exposed to perinatal inflammation, gestational age, inclusion criteria, exclusion criteria, EEG settings, time of EEG recording, statistical methods, funding sources.

### Study risk of bias assessment

The Joanna Briggs Institute (JBI) Critical Appraisal Checklist for Analytical cross-sectional Studies for use in systematic reviews was used to assess the risk of bias in the included studies^21^. Two reviewers (AG and CMS) independently assessed the risk of bias in the included studies. Any disagreements during the risk of bias assessment were discussed with a third reviewer (BHW) to make the final decision.

### Synthesis method

A narrative approach was used to synthesise the data, using the guidelines of the Economic and Social Research Council (ESRC) Methods Programme^22^, in line with the recommendations from the Cochrane Collaboration^23^. A meta-analysis of effect measure was not performed as the two included studies were insufficiently similar^22^. Each study was described in a systematic way using narrative descriptions and tabulation. All data came from the primary reference for each included study.

## Results

### Study selection

**Fig. 1:**
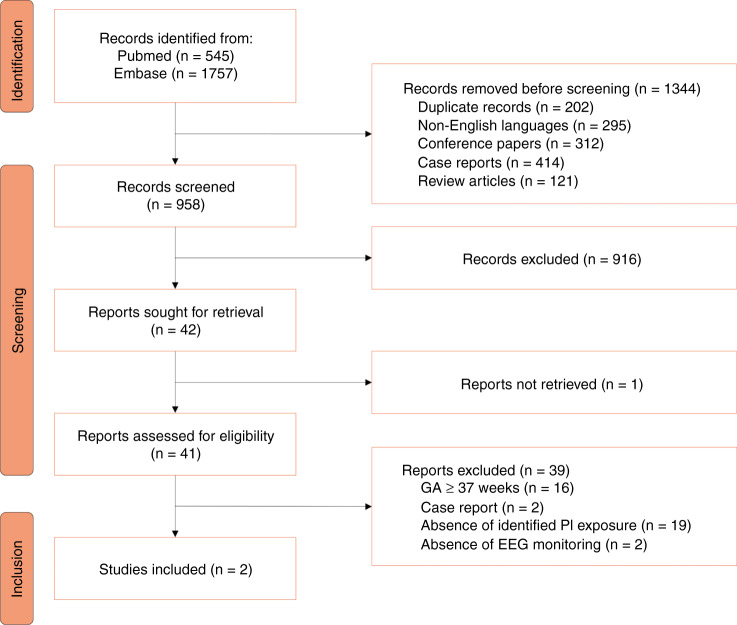
Flow diagram. *EEG* electroencephalography, *GA* gestational age, *PI* perinatal inflammation.

The study selection process is summarised in Fig. 1. A total of 2302 records were identified in databases and were exported to EndNote. After removal of duplicate records (*n* = 202), non-English language records (*n* = 295), conference paper records (*n* = 312), case report records (*n* = 414), and review article records (*n* = 121), 958 records were screened. Then, 41 full-text documents were assessed for eligibility, and finally, two studies were included in the review^24,25^.

### Study characteristics

The two included studies were the cross-sectional studies from Natalucci et al.^24^ and Paz-Levy et al.^25^ Their characteristics are detailed in Table 1.

**Table 1:** Characteristics of included studies.

Characteristics	Natalucci et al.^24^	Paz-Levy et al.^25^
Study design	Cross-sectional study	Cross-sectional study
Study location	NICU of Zurich University Hospital, Switzerland	NICU of Soroka University Medical Centre, Israel
N of centres	1	1
Duration of study (months)	January 2009–December 2011 (24)	2008–2010 (24)
Inclusion criteria	GA <32 weeks	GA ≤28 weeks; aEEG monitoring initiated <6 h of age; placental specimen available for histologic examination
Exclusion criteria	Cerebral haemorrhage; periventricular leukomalacia; ventriculomegaly; arterial hypotension necessitating therapy during aEEG monitoring; chromosomal or congenital anomalies; inborn errors of metabolism	Major central nervous system anomalies; chromosomal abnormalities
N of infant included	96	34
Perinatal inflammation	Histological chorioamnionitis; culture-proven EONI	Histological chorioamnionitis with and without funisitis
EEG settings	aEEG; one channel corresponding to P3-P4	aEEG; one channel corresponding to P3-P4
Time of EEG recording	From <24 h to days 3–4	From <6 h to 72 h
Evaluated EEG periods	Analyses performed on each 3-h artefact-free period with impedance ≤10 kOhm.	Analyses performed on each ten-minute segment of the aEEG recording, displayed in 3 timepoints corresponding to day 1, day 2, and day 3.
EEG analyses	Visual analysis using the Burdjalov scoring system for brain maturity evaluation: continuity, cycling, amplitude of the lower border, and bandwidth of the aEEG amplitude^26^. Mechanical quantitative analysis: measure of the mean maximum and minimum aEEG amplitude values^43^.	Visual analysis using the Olischar classification: isoelectric, burst suppression, low discontinuous, high discontinuous, continuous, and artefact^28^; record of daily presence of cyclicity and seizure activity.

*aEEG* amplitude-integrated EEG, *EEG* electroencephalography, *EONI* early-onset neonatal infection, *GA* gestational age, *n* number, *NICU* neonatal intensive care unit.

### Risk of bias in studies

**Fig. 2:**
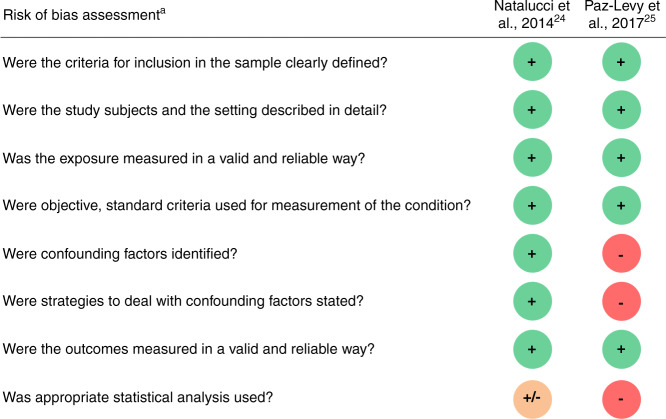
Risk of bias assessment of included studies. − no, + yes, +/− unclear. ^a^According to the Joanna Briggs Institute (JBI) Critical Appraisal Checklist for Analytical Cross-Sectional Studies^21^.

The study from Natalucci et al.^24^ included 96 (74%) neonates and presented a moderate risk of bias, due to statistical analysis methodology. Sixteen covariates were included in the multivariate regression model. All these covariates were selected based on clinical relevance, and univariate analyses were not used to screen for potential adjustment covariates. Moreover, no collinearity assessment of the multivariate regression model was performed (Fig. 2).

The study from Paz-Levy et al.^25^ included 34 (26%) neonates and presented a high risk of bias due to the absence of identification of confounding factors, the absence of strategies to deal with confounding factors, the absence of multivariate analysis, and the use of the Mann-Whitney test for multiple comparisons without p-value correction (Fig. 2).

### Results of individual studies and synthesis

The individual results of included studies are detailed in Table 2. The two included cross-sectional studies included 130 preterm children born less than 32 weeks of GA from 2008 and 2011 with a continuous one-channel aEEG assessment during the first four days of life^24,25^. Amongst them, 39 (30%) infants were exposed to histological chorioamnionitis.

**Table 2:** Summary of included studies.

Studies	Perinatal inflammation (*n*)	Infants included	Mean GA	Time of EEG recording	EEG findings	
					Visual analysis	Mechanical quantitative analysis
Natalucci et al.^24^	Histological chorioamnionitis (18); culture-proven EONI (3)	96	29.5 (range 24.4 to 31.9) weeks	From a mean (range) age of 13 (1–21) h to 90 (72–96) h	*Univariate analysis*:^a^ chorioamnionitis trended to be associated with a decrease in total maturity score of −1.24 (−2.63 to 0.16, *p* = 0.08); no association between chorioamnionitis and cycling subscore; no association between culture-proven EONI and aEEG findings.	*Univariate analysis*:^a^ chorioamnionitis was associated with a decrease in maximum aEEG amplitude of −2.33 (−4.45 to −0.21, *p* = 0.03) and in minimum aEEG amplitude of −0.65 (−1.21 to −0.10, *p* = 0.02); no association between culture-proven EONI and aEEG findings.
					*Multivariate analysis*:^b^ no independent association between chorioamnionitis and total maturity score or cycling subscore.	*Multivariate analysis*:^a,b^ chorioamnionitis trended to be independently associated with a decrease in maximum aEEG amplitude of −2.10 (−4.37 to –0.16, *p* = 0.07); no independent association between chorioamnionitis and changes in minimum aEEG amplitude.
Paz-Levy et al.^25^	Histological chorioamnionitis (21)	34	26.1 (SD 1.3) weeks	From <6 h to 72 h of age	*Univariate analysis*: chorioamnionitis with funisitis was associated with a higher incidence of absent sleep-wake cycling pattern during day 1 (50% vs. 0 to 19%, *p* = 0.037); no association between chorioamnionitis and the incidence of absent sleep-wake cycling pattern found during days 2 and 3; no association between chorioamnionitis and daily percentage of depressed aEEG pattern.	ND
					*Multivariate analysis*: ND	

All studies were cross-sectional studies performed with amplitude-integrated EEG using one channel corresponding to P3-P4.

*aEEG* amplitude-integrated EEG, *EEG* electroencephalography, *EONI* early-onset neonatal infection, *GA* gestational age, *n* number, *ND* not done, *SD* standard deviation, *SNAPPE-II* score for neonatal acute physiology with perinatal extension-II.

^a^Results displayed as slope coefficients with (95% confidence interval).

^b^Covariates included in the multivariate analysis were GA, postnatal age, male sex, small-for-GA status, histological chorioamnionitis, complete antenatal corticosteroids, arterial umbilical cord pH, 5-min Apgar score, the highest decile for GA of the SNAPPE-II, mechanical ventilation, surfactant therapy, culture-proven EONI, hypoglycaemia, morphine, caffeine, and indomethacin. Culture-proven EONI was not assessed in multivariate analysis^24^.

In their cross-sectional study including 96 neonates born before 32 weeks of GA, Natalucci et al. identified 18 infants with histological chorioamnionitis and three infants with culture-proven EONI^24^. After univariate analysis, histological chorioamnionitis was associated with a decrease in maximum aEEG amplitude (slope coefficient −2.33; 95% CI −4.45 to −0.21; *p* = 0.03) and in minimum aEEG amplitude (slope coefficient −0.65; 95% CI −1.21 to −0.10; *p* = 0.02) in the first days of life^24^. Histological chorioamnionitis was not associated with changes in total maturity score and cycling subscore according to the Burdjalov classification^26^. After multivariate analysis, histological chorioamnionitis tended to be associated with a decrease in maximum aEEG amplitude (slope coefficient −2.10; 95% CI −4.37 to –0.16; *p* = 0.07) in the first days of life^24^. Histological chorioamnionitis was not independently associated with changes in minimum aEEG amplitude, total maturity score, and cycling subscore after multivariate regression^24^. The association of culture-proven EONI with aEEG findings was not significant after univariate analysis, and not assessed in multivariate analysis because of the small number of patients with culture-proven EONI (Table 2)^24^.

In their cross-sectional study including 34 neonates born before 28 weeks of GA, Paz-Levy et al. identified eight infants with foetal amniotic fluid infection (AFI) and 13 infants with maternal AFI^25^, which are equivalent to histological chorioamnionitis with and without funisitis, respectively^27^. Histological chorioamnionitis with funisitis – but not histological chorioamnionitis without funisitis – was associated with a higher incidence of absent sleep-wake cycling pattern during the first day of life^25^. No association between histological chorioamnionitis and the incidence of absent sleep-wake cycling pattern were found during second and third days of life^25^. Additionally, no association between histological chorioamnionitis and the daily percentage of depressed aEEG pattern according to the Olischar classification^28^ was found over the first three days of life (Table 2)^25^.

## Discussion

The two studies included in this systematic review found that perinatal inflammation was associated with an altered aEEG background on univariate analysis, which could suggest an impaired EEG maturation in the first days of life among premature infants (Table 2)^24,25^. However, these results should be interpreted with caution because of the small number of included studies and their heterogeneity.

In the cross-sectional study from Natalucci et al., very preterm neonates exposed to histological chorioamnionitis displayed lower aEEG amplitudes in the first days of life^24^. In this sample of 96 very preterm infants, only 18 had chorioamnionitis. The exclusion of neonates with cerebral haemorrhage and white matter injury could explain a lower rate of infants with histological chorioamnionitis than expected^29^, as chorioamnionitis is a risk factor of neonatal brain injury in preterm children^7,8^. There was no distinction between chorioamnionitis with or without funisitis, unlike in the Paz-Levy et al. study^25^. After multivariate analysis, histological chorioamnionitis trended to be associated with a lower maximum aEEG amplitude^24^. However, the extensive number of 16 covariates included in the multivariate analysis could have introduced statistical issues such as collinearity, that may have removed any factor that had less of an impact than GA. The association of culture-proven EONI with aEEG findings could not be assessed in multivariate analysis due to the small number of patients with culture-proven EONI^24^. The exclusion of neonates with hypotension necessitating therapy during aEEG monitoring could explain the small number of patients with culture-proven EONI.

In the cross-sectional study from Paz-Levy et al., extremely preterm neonates exposed to histological chorioamnionitis with funisitis displayed a higher rate of absent sleep-wake cycling pattern during the first day of life^25^. However, these results must be considered cautiously as preterm neonates tend not to display clear sleep-wake cycling on EEG at this stage^30^, and this study presented a high risk of bias.

The included studies only assessed histological chorioamnionitis^24,25^. No study assessing clinical chorioamnionitis was identified. Rather than histological chorioamnionitis^9,31^, clinical chorioamnionitis is an independent risk factor of CP and other neurodevelopmental issues in preterm children^9–11^. Furthermore, the included studies were performed with aEEG using only one channel corresponding to P3-P4^24,25^. One-channel aEEG provides limited measures, particularly in preterm neonates who do not display clear sleep-wake cycling on EEG before 29 weeks of GA^30^.

The results described in the included studies are in line with those of Wikström et al., who found a positive correlation of cord blood TNF-α with minimum and maximum aEEG interburst intervals during the first 72 h of life in a cohort of 16 infants born before 28 weeks of GA (*r*_s_ = 0.595; *p* = 0.025)^32^. This study did not meet the inclusion criteria because it did not identify perinatal inflammation exposure as defined in our pre-established protocol. Specifically, while the cord blood was analysed for several cytokines, there is no information on the presence or absence of chorioamnionitis and EONI^32^. Cord blood TNF-α concentration is not specific to perinatal inflammation and has been associated with multiple pathologies such as maternal obesity, gestational diabetes mellitus, and perinatal asphyxia^33–35^. Also, the study published by Lee et al. describing a lower mean aEEG Burdjalov maturation score^26^ at 35 weeks of postmenstrual age in preterm infants exposed to systemic inflammation *versus* control (9.5 *versus* 8; *p* = 0.017) was not included in our systematic review^36^. In this study, the group exposed to systemic inflammation included infants with EONI, late-onset neonatal infection, and necrotising enterocolitis, without subgroup analyses for each exposition^36^.

Neonatal EEG has been shown to be relevant in other neonatal inflammation exposures, such as neonatal meningitis and late-onset neonatal infection^37–40^. Non-specific EEG background abnormality and electrographic seizures are associated with adverse outcome in neonatal meningitis, making EEG a useful tool to identify infants at higher risk for poor developmental outcome^37–39^. Interestingly, positive Rolandic sharp waves have been associated with white matter injury in neonatal meningitis^37^. Such white matter injury has also been independently associated with perinatal inflammation exposure in preterm infants^41^. Neonatal EEG has been proposed as a marker of sepsis-associated encephalopathy (SAE) in late-onset neonatal infection by Helderman et al.^40^. The authors described an association between late-onset neonatal infection and the presence of aEEG burst suppression patterns (odds ratio 2.4; 95% CI 1.2–4.8; *p* = 0.01) but found no difference for aEEG Burdjalov maturation scores^26^ in preterm infants with late-onset neonatal infection *versus* control^40^. The features associated with SAE in adults include predominant delta waves, triphasic waves, and the presence of burst suppression^42^.

In addition to the included clinical studies, seven experimental studies assessed the effect of perinatal inflammation on EEG in preterm sheep models^12–18^. Exposure to perinatal inflammation led to a deterioration of EEG maturation in six of these studies, characterised by a decreased EEG amplitude^12,13^, a decreased EEG power^14,15^, and an increased delta frequency^16,17^. One study found an increased EEG mean frequency four days after progressive infusion of LPS^18^. These preclinical studies are summarised in Table 3. The lack of clinical data evaluating the association of perinatal inflammation exposure with EEG findings in preterm children, despite the link existing between perinatal inflammation and impaired EEG maturation in preclinical studies, paves the way to future clinical studies investigating the association of perinatal inflammation exposure with conventional EEG maturation in preterm children.

**Table 3:** Summary of the seven experimental studies assessing the effect of perinatal inflammation on EEG in preterm animal models.

Studies	Perinatal inflammation	*N* exposed	*N* controls	Time of EEG recording	Duration of EEG recording	Percentage of full-term gestation	EEG findings in PI animals
Bennet et al.^12^	OK-432; 0.1 mg IP	6	7	From 12 h before to 7 days after inoculation	180 h	70%	Decreased EEG amplitude between 4 and 7 h (15.4 ±1.1 dB vs. 18.3 ±1.1 dB, *p* < 0.005); no differences in EEG spectral edge; one foetus developed electrographic seizures.
Gavilanes et al.^16^	LPS; 10 mg IA	6	7	2 days after infusion	5 min	70%	No differences in EEG frequency or amplitude.
		5	7	14 days after infusion	5 min	70%	Increased percentage of delta frequency (59.50% ±2.15 vs. 51.00% ±1.89, *p* < 0.05); no differences in EEG amplitude.
Dean et al.^13^	LPS; 200 ng/kg IV	9	11	From 12 h before to 10 days after infusion	252 h	70%	Decreased EEG amplitude between 3 and 4 h (11.2 ±0.5 µV vs. control group, 14.3 ±1.3 µV, *p* < 0.05), and from 198 h to the end of recording (240 h, *p* < 0.05); no differences in EEG spectral edge.
Keogh et al.^17^	LPS; continuous low-dose infusion 5 days IV^a^	6	9	From 12 h before to 10 days after infusion	252 h	70%	Decreased spectral edge frequency with a reduced proportion of alpha and beta power, and a higher proportion of delta power from day 6 to the end of recording (day 10, *p* < 0.05).
Galinsky et al.^14^	LPS; continuous low-dose infusion 5 days IV^b^	8	7	From 12 h before to 10 days after infusion	252 h	70%	Decreased EEG power between 49 and 53 h and at 57 h (1–5 and 9 h after the first LPS bolus, *p* < 0.05); no differences in EEG spectral edge.
Galinsky et al.^18^	LPS; progressive infusion 5 days IV^c^	7	6	From 12 h before to 10 days after infusion	252 h	70%	Increased EEG mean frequency between days 4 and 7 (12 ± 0 Hz vs. 11 ± 0 Hz, *p* < 0.05); no differences in EEG power.
Kelly et al.^15^	LPS; progressive infusion 3 days IV^d^	8	9	From 24 h before to 4 days after infusion	120 h	85%	Decreased EEG power from 3 h to 6 h and at 10 h, from 40 h to 44 h, from 72 h to 78 h, from 82 h to the end of recording (96 h, *p* < 0.05); no differences in EEG frequency.

All studies were performed on foetal sheep with conventional EEG.

*EEG* electroencephalography, *LPS* lipopolysaccharide from E. coli, *OK-432* killed Su-strain of Streptococcus pyogenes, *IA* intra-amniotic, *IP* intrapleural, *IV* intravenous, *n* number, *PI* perinatal inflammation.

^a^100 ng/kg over 24 h then 250 ng/kg/24 h for 4 days.

^b^100 ng/kg over 24 h then 250 ng/kg/24 h for 4 days plus 1 μg boluses at 48, 72, and 96 h.

^c^200 ng/kg over 24 h, then doubled every 24 h for 4 days.

^d^300 ng/kg, 600 ng/kg, and 1200 ng/kg at 0 h, 24 h, and 48 h, respectively.

A limitation of the review processes is the exclusion of non-English language records, which could lead to a selection bias. However, they represented 12.8% of identified records. Another limitation is the small number of included studies, representing a limited number of preterm children. Because of the few studies included and their heterogeneous nature, a meta-analysis could not be performed.

## Conclusion

There is insufficient clinical data to determine an association of perinatal inflammation with EEG findings in preterm infants. The two studies included in this systematic review reported inconsistent findings and were limited to aEEG analysis. No study using conventional multi-channel EEG assessment was identified. Preclinical data suggest that perinatal inflammation exposure could impair EEG maturation and that quantitative analysis of EEG may be very helpful. Further clinical studies assessing the association of perinatal inflammation with the EEG in preterm children are needed.

## Supplementary information

Supplementary Information

## Data Availability

The datasets are available from the corresponding author on reasonable request.
